# Long-term pulmonary and neurodevelopmental impairment in a fetal growth restriction rabbit model

**DOI:** 10.1038/s41598-023-48174-6

**Published:** 2023-11-28

**Authors:** Ignacio Valenzuela, Yannick Regin, Andre Gie, David Basurto, Doaa Emam, Marianna Scuglia, Katerina Zapletalova, Marnel Greyling, Jan Deprest, Johannes van der Merwe

**Affiliations:** 1 Department of Development and Regeneration, Cluster Woman and Child, Group Biomedical Sciences, KU Leuven, Herestraat 49, Box 805, B-3000 Leuven, Belgium; 2 Department of Paediatrics and Child Health, Faculty of Medicine and Health Sciences, Stellenbosch University and Tygerberg Hospital, Cape Town, South Africa; 3 Department of Obstetrics and Gynaecology, University Hospitals Tanta, Tanta, Egypt; 4 Developmental Biology and Cancer Research and Teaching Department, Great Ormond Street Institute of Child Health, University College London, London, UK; 5 Third Faculty of Medicine, Institute for the Care of Mother and Child, Charles University, Prague, Czech Republic; 6 Department of Obstetrics and Gynecology, Division Woman and Child, University Hospitals Leuven, Herestraat 49, 3000 Leuven, Belgium

**Keywords:** Disease model, Preclinical research, Experimental models of disease

## Abstract

Fetal growth restriction (FGR) remains one of the main obstetrical problems worldwide, with consequences beyond perinatal life. Animal models with developmental and structural similarities to the human are essential to understand FGR long-term consequences and design novel therapeutic strategies aimed at preventing or ameliorating them. Herein, we described the long-term consequences of FGR in pulmonary function, structure, and gene expression, and characterized neurodevelopmental sequelae up to preadolescence in a rabbit model. FGR was induced at gestational day 25 by surgically reducing placental blood supply in one uterine horn, leaving the contralateral horn as internal control. Neonatal rabbits born near term were assigned to foster care in mixed groups until postnatal day (PND) 21. At that time, one group underwent pulmonary biomechanical testing followed by lung morphometry and gene expression analysis. A second group underwent longitudinal neurobehavioral assessment until PND 60 followed by brain harvesting for multiregional oligodendrocyte and microglia quantification. FGR was associated with impaired pulmonary function and lung development at PND 21. FGR rabbits had higher respiratory resistance and altered parenchymal biomechanical properties in the lungs. FGR lungs presented thicker alveolar septal walls and reduced alveolar space. Furthermore, the airway smooth muscle content was increased, and the tunica media of the intra-acinar pulmonary arteries was thicker. In addition, FGR was associated with anxiety-like behavior, impaired memory and attention, and lower oligodendrocyte proportion in the frontal cortex and white matter. In conclusion, we documented and characterized the detrimental pulmonary function and structural changes after FGR, independent of prematurity, and beyond the neonatal period for the first time in the rabbit model, and describe the oligodendrocyte alteration in pre-adolescent rabbit brains. This characterization will allow researchers to develop and test therapies to treat FGR and prevent its sequelae.

## Introduction

During pregnancy, the intrauterine environment determines the developing fetus’ phenotype for the neonatal period and beyond. As proposed by Barker et al.^1,2^, suboptimal intrauterine conditions can lead to a higher risk of developing diseases later in life, a phenomenon also known as ‘fetal programming’. Fetal growth restriction (FGR), the failure to attain the full genetic growth potential during fetal life, is a common clinical scenario of a suboptimal intrauterine environment and it is the single largest contributing factor to perinatal mortality in structurally normal fetuses^3,4^. Furthermore, children and adolescents who had FGR are more likely to develop asthma and poorer pulmonary function^5,6^, as well as neurodevelopmental deficits and learning impediments^7–10^. Ultimately, even in adulthood, FGR is associated with worse lung function, higher respiratory morbidity, neuropsychiatric disorders, and lower cognitive function^11–15^.

Several animal models have been used to mimic human FGR and its consequences^16^. Animal models enable researchers to overcome the evident ethical constrains in studying human subjects, while allowing to control variables to limit noise and bias. While there is no such thing as a perfect model, the similarities in the development of key organ systems (respiratory, neurological, cardiovascular) determine a model’s translational value. The rabbit has been used to model FGR because it mimics human placental structure and organ development closer than other frequently used animals such as rats, mice, and sheep. The rabbit’s discoid placenta is hemochorial in structure and its two cellular layers between maternal and fetal blood closes the gap between the three-layer murine and the one-layer human placenta^17^. Additionally, the development of brain and lungs, which have been shown to be affected by FGR, also resembles that of humans. Alveolarization is a perinatal process in rabbits, unlike in rodents, so that at birth both the rabbit and the human are at the terminal air sac stage^18^. Neurogenesis onset occurs in the first trimester in the rabbit brain, and white matter maturation is a perinatal process that continues throughout the first year of life in both the human and rabbit, unlike the more precocial sheep and more altricial mouse and rat^19,20^. These practical considerations make the rabbit a valuable translational model to study the short and long-term effects of prenatal conditions and, eventually, test perinatal interventions^16^.

We have previously characterized the neurodevelopmental and pulmonary consequences of FGR in the uteroplacental vessel ligation (UPVL) rabbit model at postnatal day 1 (PND 1)^21^. We described the altered neurobehavior, regional neuropathology, and neurostructure in MRI associated with FGR. Others have found neurobehavioral and multiregional neurostructural alterations in the long-term using the same model^22–24^. However, there is currently no data on the pulmonary sequelae after the neonatal period in rabbit models. Herein, we investigated the mid and long-term effects of FGR in the lungs and brain by describing pulmonary function, microarchitecture, and gene expression. In addition, we longitudinally assessed neurobehavior and described its associated multiregional neuropathology.

## Results

### FGR is associated with higher mortality and persistently lower body weight

**Figure 1:**
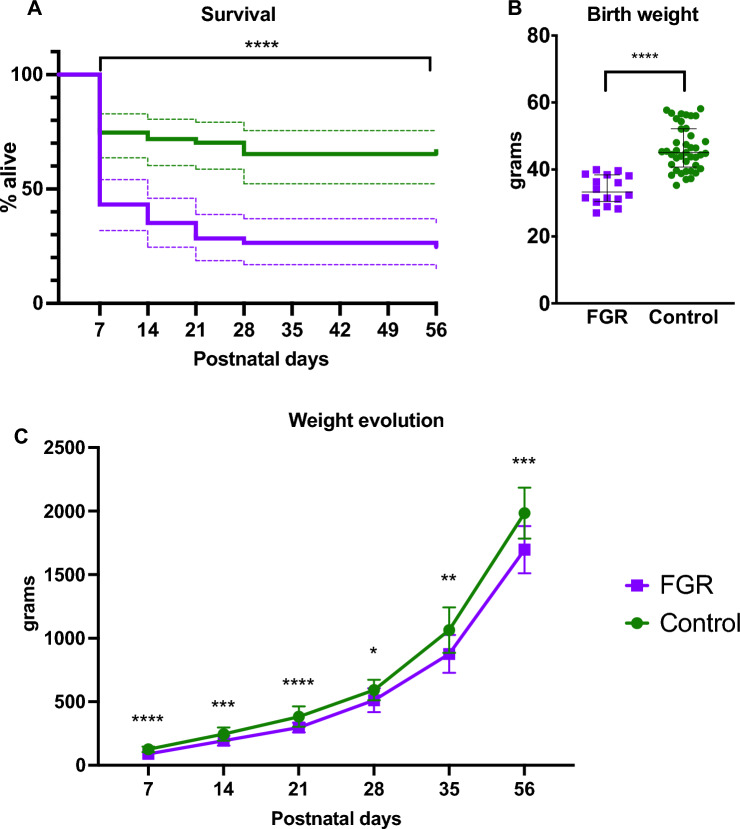
Postnatal survival and biometrics of FGR and control rabbits. (**A**) Postnatal survival of 155 rabbits from 30 litters. Controls without littermates were excluded from further analysis. (**B**) Birth weight of animals that survived until last assessment time point (n, FGR = 16; control = 41). (**C**) Weight evolution of animals that survived until last assessment time point. Data were analyzed using a linear mixed-effects model. Values as mean ± SD, **p < 0.01; ***p < 0.001; ****p < 0.0001.

Thirty dams delivered 237 live kittens, 104 from the ligated horns and 133 from the control horns. UPVL resulted in significantly lower survival at birth (51.48 vs. 85.80%; p < 0.0001). One-hundred and fifty-five neonates (75 FGR and 80 controls) were randomly assigned to 18 foster dams. In those, FGR was associated with lower birth weight (32.00 ± 5.54 vs. 43.69 ± 8.16 g; *p* < 0.0001), increased neonatal mortality in the first 3 weeks, and significantly decreased body weight until preadolescence (Fig. 1, Table S2). Sixteen FGR kittens (21%) survived until their final assessment time point, and 52 controls (65%) survived until final assessment time point or until no more FGR kittens remained in their litters and had to be excluded from further analysis. Fifty-seven kittens (16 FGR, 41 controls) were considered for pulmonary or brain assessment. Among these animals, FGR was also associated with a lower birth weight (33.86 ± 4.31 vs. 46.27 ± 6.62 g; *p* < 0.0001).

### FGR is associated with altered parenchymal tissue properties and airway mechanics at PND 21

**Figure 2:**
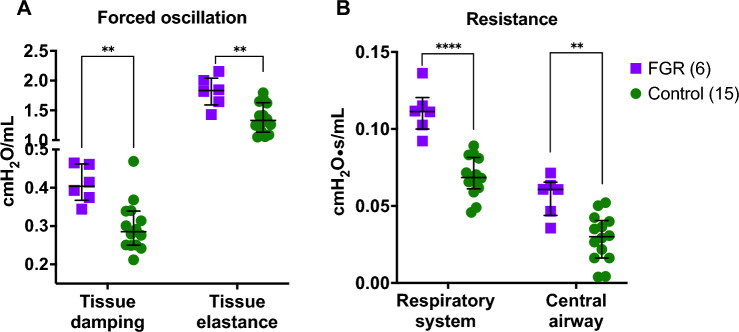
Pulmonary functional tests at postnatal day 21. (**A**) Results from forced oscillation maneuvers, showing increased tissue damping and elastance in lung parenchyma from FGR rabbits. (**B**) Increased resistance in the respiratory system, and in the conducting airway in the FGR group. Data were analyzed using a linear mixed-effects model. Values as mean ± SD, **p < 0.01; ****p < 0.0001.

Lungs from the FGR group displayed increased tissue damping (0.41 ± 0.05 vs. 0.30 ± 0.07 cmH_2_O/mL; *p* = 0.001), tissue elastance (1.82 ± 0.26 vs. 1.37 ± 0.25 cmH_2_O/mL; *p* = 0.001), respiratory system resistance (0.11 ± 0.01 vs. 0.07 ± 0.01 cmH_2_O·s/mL; *p* < 0.0001) and central airway resistance (0.056 ± 0.01 vs. 0.029 ± 0.01 cmH_2_O·s/mL; *p* = 0.001) when compared to controls (Fig. 2).

Deep inflation and pressure–volume maneuvers showed reduced static compliance (2.22 ± 0.47 vs. 2.62 ± 0.50 mL·cmH_2_O^−1^·kg^−1^; *p* = 0.01) but comparable inspiratory capacity (34.25 ± 7.85 vs. 32.00 ± 7.03 L/g; *p* = 0.5) after correcting by body weight.

### FGR is associated with thicker alveolar walls and increased airway smooth muscle content (ASMC)

**Figure 3:**
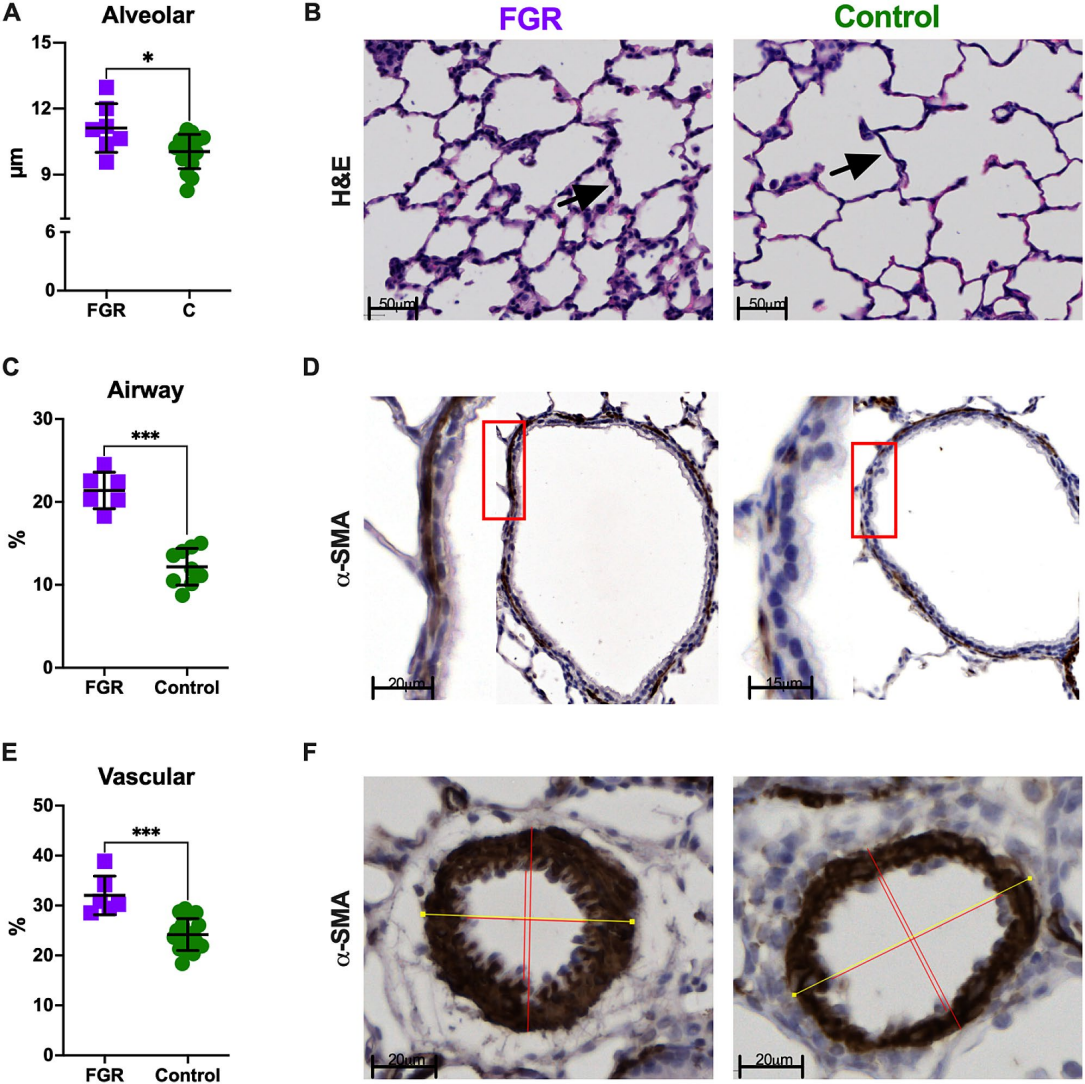
Histology in postnatal day 21 rabbit lungs (FGR = 6; controls = 10). (**A**) Alveolar morphometric data. (**B**) Representative images of histological H&E-stained slides, with arrows showing alveolar walls. (**C**) Airway smooth muscle content. (**D**) Representative α-SMA images of conducting airways, smooth muscle is dark brown. (**E**) Pulmonary arteries medial thickness. F. Representative α-SMA images of peripheric pulmonary arteries. Data were analyzed using a linear mixed-effects model. Values as mean ± SD, or median and IQR according to data characteristics. *p < 0.05; ***p < 0.001.

FGR was associated with increased alveolar wall thickness (Lmw, 11.12 ± 1.1 μm vs. 10.05 ± 0.78 μm; *p* = 0.01) decreased airspace size (Lma, 43.69 ± 3.7 μm vs. 48.01 ± 4.6 μm; *p* = 0.03), resulting in comparable alveolar size (Fig. 3). The ASMC was higher in conducting airways of FGR lungs (21.37% [19.76–23.03] vs 11.9% [10.31–14.31]; *p* = 0.0004; Fig. 3). In addition, the medial thickness of pulmonary arteries was increased in rabbits born after FGR (30.63% [29.3–35.4] vs. 23.87% [21.9–26.4]; *p* = 0.0001; Fig. 3). Expression of genes related to angiogenesis, inflammation, surfactant production and collagen production was not significantly different between groups (Supplementary materials, Table S3).

### FGR is associated with persisting neurodevelopmental impairment until preadolescence

**Figure 4:**
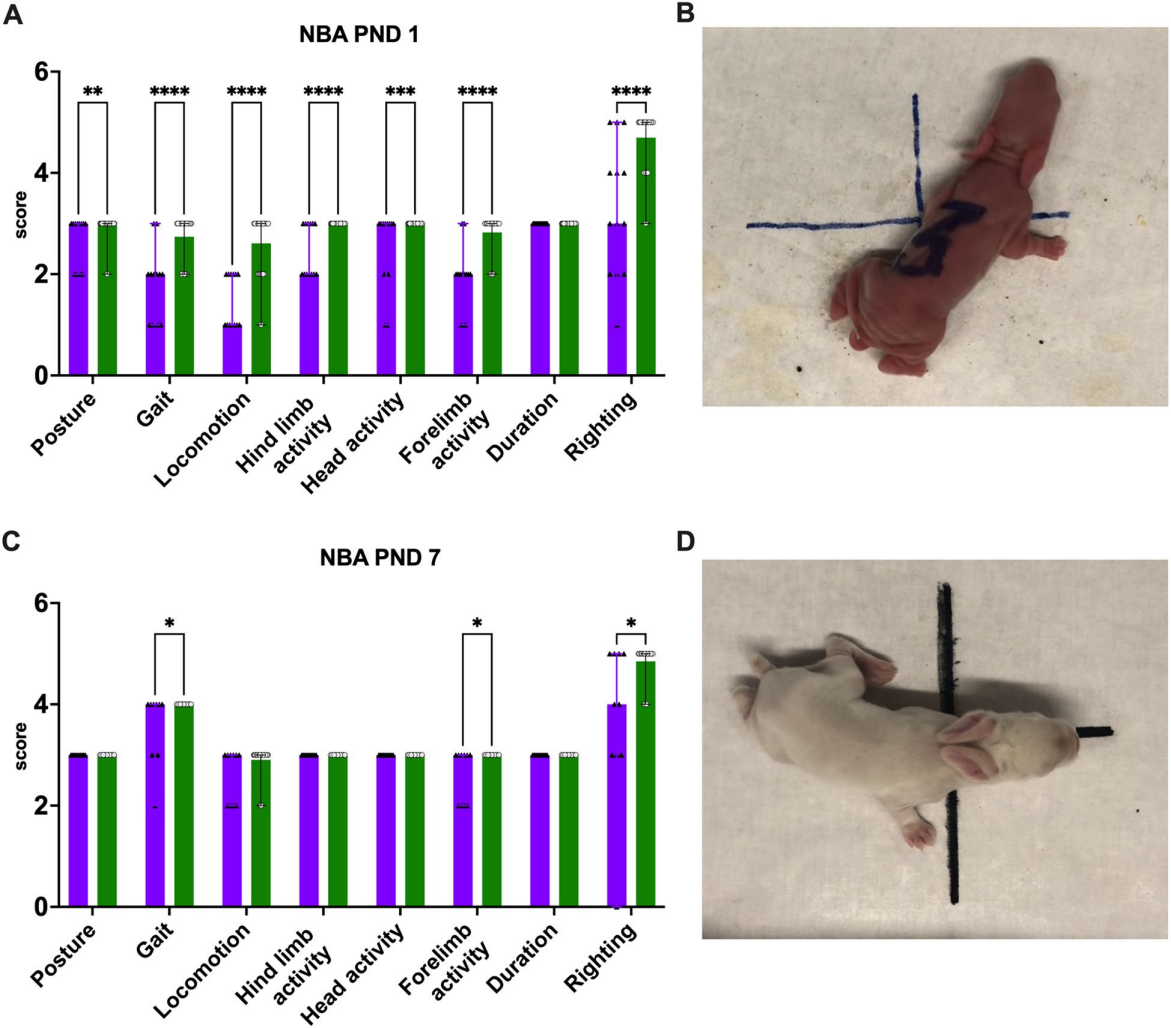
(**A**, **C**) Individual scores of neurobehavioral assessment at postnatal days 1 and 7, respectively. (**B**, **D**) Representative images of kittens at each gestational age. FGR n = 10; controls n = 22. Values as median and IQR, *p < 0.05; **p < 0.01; ***p < 0.001; ****p < 0.0001.

FGR rabbits had lower scores in both motoric and sensorial tests at early neurobehavioral assessment (NBA), as displayed in Fig. 4. At PND 1, FGR kittens had abnormal posture, gait, locomotion, and limb/head activity, but the amount of time they remained active during assessment (activity duration) was no different from controls. Additionally, FGR was associated to hampered cranial nerves activity and righting reflex. At PND 7, gait, forelimb activity, and righting reflex remained compromised in FGR rabbits (Fig. 4).

At PND 21–25, rabbits from the FGR group presented a lower total travelled distance in the open field test (OFT) and a significantly lower discriminatory index (DI) in the novel object recognition test (NORT) as summarized in Table 1. In the same subjects evaluated at PND 56–60, FGR was associated with increased total distance in the OFT, more escape attempts, and less time lying down, in addition to a lower DI in NORT (Table 1).

**Table 1:** Neurobehavioral assessment at week 4 and 9.

	Postnatal days 21–25			Postnatal days 56–60		
	FGR	Control	*p* value	FGR	Control	*p* value
**Open field test**						
Total distance (m)	9.8 ± 3.5	13.5 ± 4.8	0.04	16.9 ± 9.0	10.5 ± 7.6	0.01
Time in starting area (%)	8.6 ± 7.6	14.8 ± 15.7	0.24	7.0 ± 10.7	6.2 ± 4.7	0.79
Time in central area (%)	9.7 ± 9.2	9.4 ± 7.9	0.93	17.7 ± 16.7	18.9 ± 25.3	0.89
Time in corners (%)	90.1 ± 9.2	90.5 ± 7.9	0.91	82.3 ± 16.7	81.0 ± 25.3	0.89
Time self-grooming (%)	47.2 ± 41.7	64.2 ± 65.5	0.49	19.0 ± 26.2	31.2 ± 56.2	0.52
Escape attempts (n)	2.7 ± 2.3	2.0 ± 1.9	0.43	2.9 ± 1.5	1.5 ± 1.7	0.03
Times rearing (n)	5.3 ± 4.9	4.6 ± 3.4	0.69	6.9 ± 7.6	3.2 ± 4.9	0.11
Time lying down (s)				0 (32.5)	120 (185)	0.02
**Novel object recognition test**						
Discriminatory index	− 0.23 ± 0.59	0.73 ± 0.27	< 0.0001	− 0.29 (0.34)	0.7 (0.57)	0.001
** T-maze test**						
Failure to choose (%)	19.23 ± 18.13	35.71 ± 25.7	0.05	0 (43.5)	0 (54.0)	0.59
Spontaneous alternation (%)	80.62 ± 25.2	68.48 ± 31.64	0.25	55.5 ± 39	81.5 ± 29.8	0.07

FGR n = 10; controls n = 22. Data as mean ± SD or median (IQR).

### FGR is associated with lower multiregional oligodendrocyte proportions in cerebral white matter tracts at PND60

**Figure 5:**
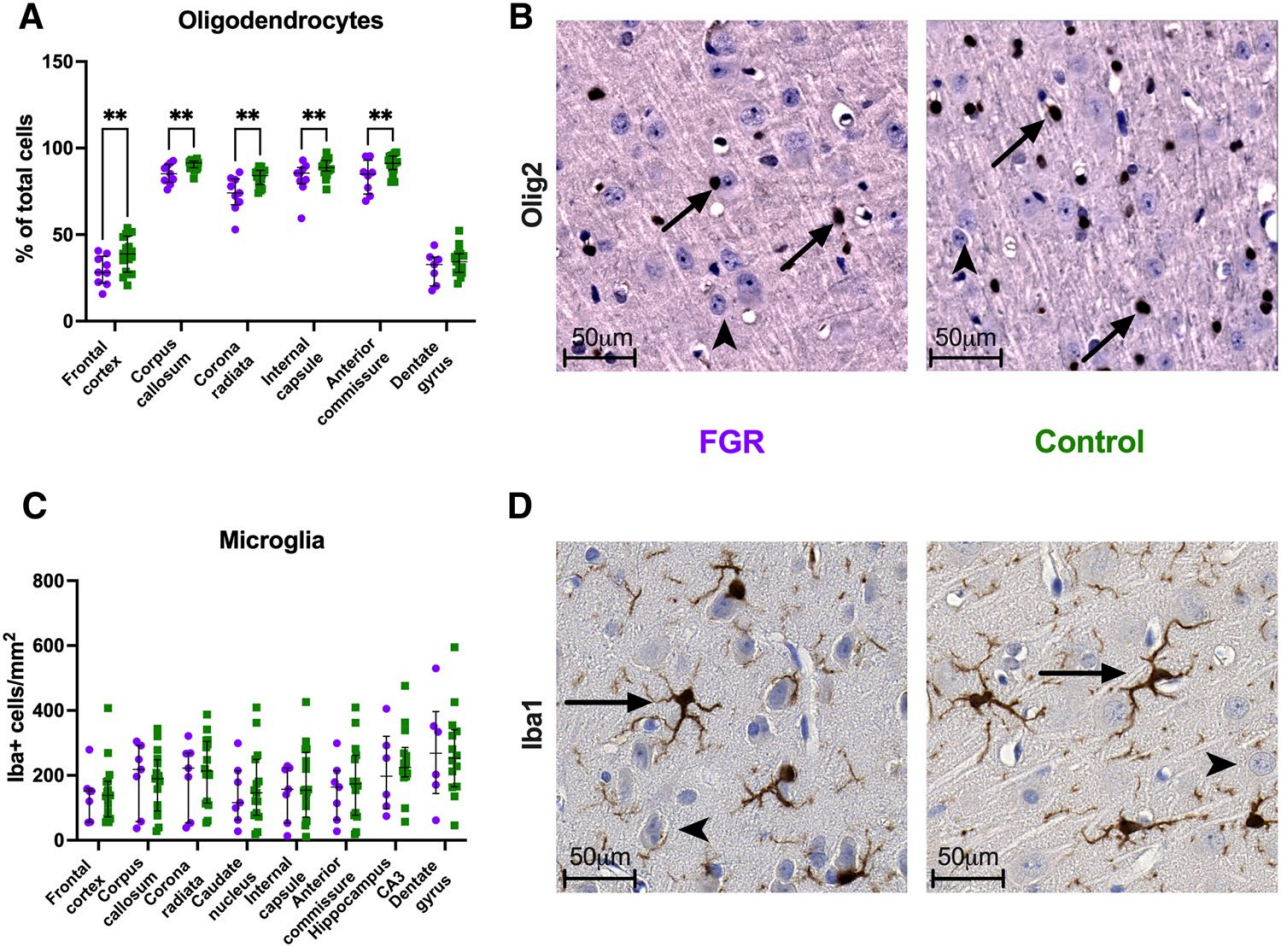
Neuropathology in postnatal day 60 rabbit brains. (**A**) Percentage of oligodendrocytes in front and midbrain structures. (**B**) Representative images from frontal cortex, showing olig2 + cells (arrows) and neurons (arrowheads) from FGR and control brains. (**C**) Density of microglia in front and midbrain structures. (**D**) Representative images from the frontal cortex, showing Iba1 + cells (arrows) and neurons (arrowheads) from FGR and control brains. CA3: cornu ammonis 3. Data were analyzed using a linear mixed-effects model. Values as mean ± SD, **p < 0.01.

Preadolescent brains from FGR rabbits had a significantly lower proportion of oligodendrocytes in the frontal cortex (FC, *p* = 0.006), corpus callosum (CC, *p* = 0.001), corona radiata (CR, *p* = 0.001), internal capsule (IC, *p* = 0.007), and anterior commissure (AC, *p* = 0.006) (Fig. 5). The density of microglia (sign of acute neuroinflammation) in both white and grey matter structures, both in the front and midbrain, did not significantly differ between FGR and control preadolescent brains. Exact oligodendrocyte and microglia populations can be found in Table S4.

## Discussion

This study provides insight into the long-term pulmonary consequences of FGR until PND 21, combining a multimodal functional, histological, and molecular approach in a relevant model. We describe the biomechanical properties, alveolar morphometry, and gene expression in rabbits born after FGR, and demonstrate that an early intrauterine insult has lasting effects on somatic growth, lung function and lung structure. In addition, we expanded the neurodevelopmental characterization from previous reports^22–25^ by characterizing the oligodendrocyte and microglia expression in selected brain regions at preadolescence.

At PND21, FGR is associated with disrupted pulmonary function indicative of parenchymal remodeling, as illustrated by higher peripheral tissue damping and elastance. These forced oscillation test findings of parenchymal remodeling are supported by the lower static compliance in the pressure–volume maneuver. In addition to the evidence of parenchymal dysfunction, we found signs of perturbed airway function following FGR. Rabbits in this group had higher central airway resistance indicating that FGR alters both peripheral (parenchymal) and central lung (airway) development. Consistent with this functional evidence, morphometric assessment demonstrates significant changes to alveolar and airway structure. FGR lungs showed increased alveolar wall thickness, decreased airspace size, and increased ASMC. This mimics the pulmonary response to postnatal hyperoxia earlier described in preterm appropriately grown rabbits showing thickened alveolar septa and increased ASMC, leading to increased resistance and peripheral tissue damping^26–28^. Since our previous work in newborn FGR rabbits at term showed a similar alveolar wall thickness the observed difference at a later age suggests FGR primes the lung for secondary injury, disrupting postnatal lung and airway development even further. However, inflammation, angiogenesis, surfactant, and collagen production gene expression did not demonstrate an ongoing aberrant process following FGR at PND21. This could imply that disruption of lung development occurs early in the postnatal phase following FGR or follows a mechanistic pathway that does not involve these specific genes. Our findings demonstrate that prenatal growth and development play an independent role to that of prematurity when it comes to pulmonary function and structure, possibly by prenatally priming the airway and lung parenchyma for an early secondary injury with further sequelae.

On NBA, FGR rabbits have increased levels of motoric and sensorial impairment at early assessment, develop behavior that suggests increased anxiety^29^, and show alterations in short-term memory and attention up until pre-adolescence, as previously published in the same model at PND 70^23^. At neuropathological assessment, the multiregional white matter reduction in oligodendrocyte populations confirms the MRI microstructural changes and, moreover, the hampered ability of neural progenitor cells to differentiate into oligodendrocytes precursors reported in this model at an earlier time point^23,24,30^. Future interventions for FGR may also aim at ameliorating these long-lasting neurocognitive sequelae^22,30^.

We acknowledge our study has limitations. First, rabbits that survived until the final assessment time point are most likely the strongest and healthiest of their litters, hence not completely representative; differences between FGR and control rabbits might have been underestimated. Moreover, the high postnatal mortality in the control group reflected the challenging foster care system, in which dams at times rejected the entire litter, neglecting healthy kittens as well as growth restricted ones. Secondly, this model describes the effects of FGR in animals born near-term, even though the most severe clinical cases often require earlier delivery, hence may display far more dramatic changes. Pulmonary and neurodevelopment are highly dependent on gestational age at delivery, and thus assessing near-term neonates allows us to unmask the effects of FGR from those of prematurity. Lastly, longitudinal pulmonary assessment was not feasible due to the terminal nature of the functional testing method used.

## Conclusion

We demonstrated the long-term pulmonary sequelae of FGR in a rabbit model. We documented significant airway and lung parenchyma changes that are not the result of prematurity or postnatal hyperoxia. Moreover, we confirmed previously described neurobehavioral changes up to preadolescence, characterized by increased anxiety-like behavior and impaired memory and attention. We further described the gray and white matter structural deficit in oligodendrocyte populations. Therefore, we think this rabbit model is ideal for testing efficacy and safety of interventions aiming at improving the short and long-term effects of FGR.

## Materials and methods

### Animal model

All methods were carried out in accordance with relevant guidelines and regulations. Animals were treated according to current guidelines for animal well-being, and experiments were approved by the Ethics Committee for Animal Experimentation of the Faculty of Medicine of KLU Leuven (P080/2019). Experiments are reported according to ARRIVE guidelines^31^. Time-mated rabbits (Dendermonde and New Zealand White hybrids) were housed in individual cages at 21 °C, 42% humidity, with a 12-h day/night cycle and free access to food and water. Conception day was considered day 0 of pregnancy. At gestational day (GD) 25 (full term 31.5 days), dams underwent UPVL to induce FGR. Briefly, pregnant rabbits were administered induction anesthesia with IM ketamine (35 mg/kg Nimatek®, Eurovet Animal Health BV) and xylazine (5 mg/kg XYL-M® 2%, VMD), antibiotic prophylaxis (10 mg/kg Baytril® 2.5% SC, Bayer), tocolysis (10 mg/kg Depo-Provera® SC, Pfizer), and analgesia (0.03 mg/kg Vetergesic® SC, Ceva Animal Health) prior to surgery. Anesthesia was maintained with a continuous IV infusion of ketamine (8–16 mg/kg/h) and xylazine (2.4–4.8 mg/kg/h), while monitoring vital signs. Following laparotomy, 33–50% of the vessels going to each placenta were ligated in one random horn with Vicryl® 5-0 (Ethicon Inc., Johnson & Johnson), leaving the contralateral horn as internal control. The abdomen was closed with Vicryl® 2-0 and Monocryl® 3-0 (Ethicon Inc, Johnson & Johnson) for fascia and skin, respectively. The surgical wound was infiltrated with levobupivacaine (2 mg/kg Chirocaine®, Abbvie) and sprayed with aluminium (Kela®).

Dams were monitored daily until delivery by caesarian section near term (GD 30). Following delivery, they were euthanized using IV phenytoin/pentobarbital (140 mg/kg Euthasol®, Kela). Only litters with liveborn cases and controls were included. Newborn kittens were pat dried, labeled with a permanent marker, and kept in a warmed (34 °C) and humidified (55% RH) incubator for 24 h. Four hours after birth they were stimulated to urinate, weighed, and fed a milk substitute (Day One, protein 30%, fat 50%; Fox Valley) with added probiotics (Bio-Lapis; Probiotics International) and immunoglobulins (Col-o-Cat; SanoBest).

### Foster care

Unmanipulated time-dated pregnant rabbits were housed in double cages with a nesting box (54 × 31 × 27 cm) at least 3 days prior to term and allowed to deliver spontaneously on the same day as the experimental rabbits. At PND 1 the experimental kittens were identified with subcutaneous chips and assigned to a foster dam. A mixed group (control and FGR) of up to 9 kittens from different litters was assigned for each foster dam and checked every 2 days for a period of 21 days until they were fully weaned. Rabbits undergoing pulmonary assessment were evaluated immediately after weaning (PND 21), while those undergoing neurodevelopmental assessment were housed with their foster litter in a double cage until preadolescence (PND 60), considering that puberty occurs at approximately PND 70. If all cases from one litter died, the control littermates were excluded from follow-up.

Initially, all rabbits underwent longitudinal neurodevelopmental assessment, until 34 rabbits reached PND 60. Thereafter, all rabbits underwent pulmonary evaluation until 23 rabbits reached PND 21.

### Pulmonary function testing

At PND 21, rabbits were deeply anesthetized with ketamine (35 mg/kg) and xylazine (6 mg/kg), and a tracheostomy was performed. An 18-gauge metal cannula was inserted into the trachea and secured with an airtight suture. Pressure–volume and forced oscillation maneuvers were performed using the FlexiVent system with the FlexiVent module 4 (FlexiVent 8.0; SciReq, Montreal, Canada). Kittens were ventilated with a tidal volume of 10 mL/kg at a rate of 120 breaths/min. Before lung function tests, two deep inflation maneuvers were performed until reaching a pressure of 27 cmH_2_O to maximally inflate the lungs and standardize lung volume. Both pressure–volume (inspiratory capacity, static compliance, and static elastance) and forced oscillation tests (tissue damping, tissue elastance, central airway resistance, respiratory system resistance, dynamic compliance, and dynamic elastance) were performed, as previously described^32^. The mean of three separate measurements, with a coefficient of determination > 95%, was used as a single data point for analysis.

### Histological lung assessment

Immediately after pulmonary function tests, deeply anesthetized animals were euthanized, and lungs were removed *en bloc* via thoracotomy. The right lung was snap frozen for molecular analysis, and the left lung was pressure fixed for 24 h at 25 cmH2O in 4% paraformaldehyde (PFA). After fixation, 6 sections from the superior and inferior lobe were embedded and cut as described before^33^. Alveolar morphology was measured on digitally scanned 4 µm hematoxylin and eosin-stained slides using a semi-automated, validated Fiji-plugin (ImageJ; http://fiji.sc/Fiji)^34^. For each lung, 3 slides from each of the 6 segments were analyzed (18 slides per lung). Calculations of the mean linear intercept (Lm), alveolar air space (Lma), and alveolar wall thickness (Lmw) were made as previously described^35^. ASMC and vascular morphology were evaluated using immunohistochemistry. A primary α-smooth muscle actin (α-SMA) antibody (mouse anti-human, M0851; DakoCytomation) was used in combination with a secondary goat anti-mouse antibody (115-035-044; Jackson ImmunoResearch). Aminoethyl carbazole was used as a chromogen.

ASMC was measured in 10 randomly selected airways per lung. Airways with a diameter of 150–250 µm if cut in cross section to their long axis were included. Airways with a long axis-to-short axis ratio of > 2:1 were excluded. ASMC was analyzed in QuPath 0.2.0^36^ by manually selecting the fraction of stained muscle tissue and the full perimeter of the airway. Vascular morphometry was performed examining a minimum of 10 intra-acinar pulmonary arteries per lung with an external diameter of 40–200 µm, measuring both internal and external diameter of the media to calculate the vascular medial thickness^32,37^.

### Lung gene expression

Total RNA was extracted from right lung homogenates using the RNeasy mini kit (Qiagen) and cDNA synthesized using TaqManTM reverse transcription reagents (Thermo Fisher Scientific). The expression of ANGPT2, SPB, SPC, VEGFA, VEFGR2, COL1A2, AND IL-8 was detected using Platinum SYBR Green qPCR Supermix-UDG with ROX (Thermo Fisher Scientific) for the purpose of examining angiogenesis, inflammation, and surfactant expression. Specimens were run in triplicate and normalized to the housekeeping gene HPRT (primers can be found in supplementary material, Table S1).

### Neurobehavioral assessment

Neonatal rabbits underwent a validated protocol for NBA at PND 1, 7, 21–25, and 56–60^23,38^. NBA at PND 1 was performed prior to microchip insertion, and included motor and sensorial assessment, as described before^39^. At PND 7, NBA consisted of OFT, righting reflex and cliff avoidance tests^23,40^. At PND 21 and 56, rabbits underwent five consecutive days of NBA, including an OFT, T- maze, and NORT^40^. Sessions were filmed and scored later by two observers blinded to group assignment.

### Neuropathological assessment

At PND 60 animals that previously underwent NBA were deeply anesthetized with IM ketamine (35 mg/kg) and xylazine (6 mg/kg), and transcardially perfused with 0.9% saline + heparin (100 u/mL; 3 min at 150 mL/min) followed by 4% PFA (4 min at 150 mL/min). Their brains were extracted, further immersed-fixed for 48 h, paraffin embedded, and serially sectioned at 4 µm. One set of four serial coronal sections was taken at each of the following two levels: level 1 started at the medial septal nucleus and level 2 at the hippocampal formation. One slide per level was incubated with rabbit monoclonal anti-Olig2 antibody (1:100, ab109186; Abcam) and a secondary goat anti-rabbit antibody (2337909; Jackson ImmunoResearch), and another slide per level with rabbit monoclonal anti-Iba1 antibody (1:500, 019-19741, Wako Pure Chemical Corp.) and a secondary swine anti-rabbit biotinylated antibody (E0431; Dako®). All slides were digitally scanned with the Zeiss AxioScan Z1 imaging platform (AxioScan® Slide Scanner, Carl Zeiss MicroImaging GmbH). Regional analysis was performed in the FC, CC, CR, IC, CN, AC, and hippocampus (CA3 and dentate gyrus), using the positive cell detection tool in Qupath^36^.

### Statistical analysis

Sample size calculation was performed by power analysis using the G*Power software^41^, for each group of outcomes separately (brain and pulmonary outcomes). We used a two-tailed approach, with α 0.05 and 1 − β at 0.8, and the effect sizes were drawn from differences observed in previous research with this model^21^ (details in Supplementary materials).

Data were analyzed and graphed using RStudio (RStudio: Integrated Development for R. RStudio, PBC, Boston, MA, USA) and Prism 10 for MacOS (GraphPad Prism, San Diego, CA, USA). Data distribution was checked for normality using a Shapiro–Wilk normality test and presented as mean with standard deviation or median with interquartile range, as appropriate. Survival curves were compared by Gehan-Breslow-Wilcoxon test, and all other parameters were analyzed using a linear mixed-effects model, considering the litter as a random effect and the presence of FGR as a fixed effect. Statistical analysis for gene expression was performed on ΔΔCt and fold change was used for visualization. A *p* value of < 0.05 was considered significant.

### Supplementary Information

Supplementary Information.

## Data Availability

The datasets analysed during the current study available from the corresponding author on reasonable request.

## Abbreviations

ANGPT2: Angiopoietin-2
ASMC: Airway smooth muscle content
CA3: Cornu ammonis 3
COL1A2: Collagen type I alpha 2 chain
FGR: Fetal growth restriction
GD: Gestational day
IL8: Interleukin 8
NBA: Neurobehavioral assessment
NORT: Novel object recognition test
OFT: Open field test
PFA: Paraformaldehyde
PND: Postnatal day
SPB: Surfactant protein B
SPC: Surfactant protein C
UPVL: Uteroplacental vessel ligation
VEGFA: Vascular endothelial growth factor A
VEGFR2: Vascular endothelial growth factor receptor 2
