# Supplementary material for: Long-term pulmonary and neurodevelopmental impairment in a fetal growth restriction rabbit model
Source: Sci Rep. 2023 Nov 28;13:20966. doi: 10.1038/s41598-023-48174-6 (PMC10684490; doi:10.1038/s41598-023-48174-6)
Supplement: Supplementary file 1 — Supplementary Information. [file 41598_2023_48174_MOESM1_ESM.pdf]

Long-term pulmonary and neurodevelopmental impairment in a fetal growth restriction rabbit model

Ignacio Valenzuela, Yannick Regin, Andre Gie, David Basurto, Doaa Emam, Mariana Scuglia, Katerina Zapletalova, Marnel Greyling, Jan Deprest, Hannes van der Merwe

Supplementary information

Sample size calculation (SSC)

Primary outcomes for SSC were Respiratory system resistance (Rrs) and oligodendrocytes in the prefrontal cortex (PFC), for which we used effect sizes obtained from our previous work with this model[1].

Rrs effect size was 2.16, with ICC=0.24
ODP in PFC effect size was 3.13 with ICC=0.44
A 2-tailed unpaired t-test, with an alpha error of 0.05 and power of 0.8 resulted in 7 kittens for the control group and 3 for the case group, considering a 2:1 ratio, due to the expected higher mortality in the FGR group. To correct for the fact that the kittens in one litter are not independent observations, we multiplied the result with the variance inflation factor (1+ICC[average cluster size-1]), which according to our previous paper results in 1.96. Therefore, the number of kittens in each group for pulmonary evaluation was 14 controls and 6 cases.
For neurological assessment, we considered oligodendrocyte count in the CN as the main outcome. Since we did not have data on that specific outcome from our previous work (oligodendrocytes are not yet expressed as such in the immediate postnatal period), we used oligodendrocyte precursors as a proxy. A 2-tailed unpaired t-test, with an alpha error of 0.05 and power of 0.8 resulted in 5 kittens for the control group and 3 for the case group, considering a 2:1 ratio. After multiplying for the variance inflation factor the number of kittens was 14 for the controls and 8 for the cases.

Supplementary Tables

Table S1. Primers used in this study

Gene	Position	Label	Primer sequence
IL8	I1	IL8 Rabbit FWD	CCACACCTTTCCATCCCAAAT
	I2	IL8 Rabbit REV	CTTCTGCACCCACTTTTCCTTG
SPC	E5	SP-C Rabbit FWD	CAAAGAGGCCTTGATGGAGA
	E6	SP-C Rabbit REV	ATCTCTAGGACCATCTCGGT
SPB	F5	SP-B Rabbit FWD	ACTGTCTACAGGAAGTCTG
	F6	SP-B Rabbit REV	GAAGTAGACGTCAAGCACG
ANGPT2	E3	ANGPT2 Rb FWD set 5	CCCGTGAACTTTGTCACTTA
	E4	ANGPT2 Rb REV set 5	CTCTCAACCTTGAACTCATCT
VEGFA	F1	TS rabbit VEGFA P1.FW	CTT GCT GCT CTA CCT CCA CC

	F2	TS rabbit VEGFA P1.RV	CTT TGG TCT GCA TTC ACA TTT G
VEGFR2	G1	TS rabbit VEGFR2 P3.FW	CCCCTGATTACACTACGCCC
	G2	TS rabbit VEGFR2 P3.RV	TGTAGTCTTTGCCACCCTGC
COL1A2	E1	TS rabbit COL1A2 P1.FW	TGCAGGGCTCCAATGATGTT
	E2	TS rabbit COL1A2 P1.RV	AGAATTCTTGGTCAGCGCCA

Table S2. Body weight evolution

Postnatal day	Mean of FGR	Mean of Control	Difference	SE of difference	t ratio	df	Individual p value
7	90.49	126.0	-35.49	5,347	6,638	42,82	<0,0001
14	193.3	245.2	-51.91	13,70	3,788	40,80	0,0005
21	297.0	382.9	-85.94	16,31	5,268	49,64	<0,0001
28	511.9	592.4	-80.48	32,71	2,461	17,34	0,0246
35	875.7	1063	-187.6	61,77	3,037	21,32	0,0062
56	1697	1984	-287.5	69,91	4,112	21,29	0,0005

Fixed effects (type III)	P value	P value summary	F (DFn, DFd)
Time	<0,0001	****	F (2,311, 80,44) = 1498
Group	<0,0001	****	F (1, 56) = 37,24
Time x Group	<0,0001	****	F (5, 174) = 8,396
Random effects	SD	Variance	
Subject	49,04	2405	
Residual	93,74	8788	

Table S3. Gene expression of postnatal day 21 rabbit lungs.

Gene	FGR* (n=6)	Control (n=6)	p-value	Adjusted p-value
ANGPT2	1.16±0.252	1.00	0.5619	0.9618
VEGFA	1.22±0.186	1.00	0.3048	0.8871
VEGFR2	1.21±0.446	1.00	0.6689	0.9618
SPB	0.926±0.115	1.00	0.5580	0.9618
SPC	0.977±0.091	1.00	0.8145	0.9618
IL8	0.759±0.284	1.00	0.4430	0.9464
COL1A2	1.57±0.404	1.00	0.2322	0.8427

* Fold change ± SE of difference. ANGPT2 = Angiopoietin-2; VEGFA = Vascular endothelial growth factor A; VEGFR2 = Vascular endothelial growth factor receptor 2; SPB = Surfactant protein B; SPC = Surfactant protein C; IL8 = Interleukin 8; COL1A2 = Collagen Type I Alpha 2 Chain.

Table S4. Neuropathological assessment

Parameter	FGR	Control	SE of difference	p-value	Adjusted p-value
**Olig2, % of positive cells**					
Frontal cortex	29.28	37.95	3.910	0.036330	0.006516
Corpus callosum	85.08	90.49	1.704	0.004103	0.001918
Corona radiata	73.57	82.98	2.921	0.003638	0.001918
Internal capsule	82.38	88.84	2.887	0.034932	0.007211
Anterior commissure	83.17	91.25	3.009	0.013925	0.006516
Dentate gyrus	30.71	34.90	3.995	0.307272	0.056510
**Iba1, number of cells per mm^2**					
Frontal cortex	138.9	150.2	44.56	0.775951	0.897466
Corpus callosum	193.6	180.9	47.28	0.777507	0.897466
Corona radiata	195.4	216.3	49.81	0.662551	0.897466
Caudate nucleus	142.7	163.8	47.71	0.676958	0.897466
Internal capsule	147.8	173.6	50.27	0.595013	0.897466
Anterior commissure	151.6	180.0	54.26	0.578142	0.897466
Hippocampus: CA3	211.9	239.8	70.58	0.613126	0.897466
Dentate gyrus	275.3	269.5	44.56	0.934942	0.944292

References

1 Valenzuela, I. *et al.* Placental vascular alterations are associated with early neurodevelopmental and pulmonary impairment in the rabbit fetal growth restriction model. *Scientific reports*. **12**, 19720, doi:10.1038/s41598-022-22895-6 (2022).
